# *In vivo* SELEX of single-stranded domains in the HIV-1 leader RNA

**DOI:** 10.1186/1742-4690-10-S1-P91

**Published:** 2013-09-19

**Authors:** Nikki van Bel, Atze T Das, Ben Berkhout

**Affiliations:** 1 Laboratory of Experimental Virology, University of Amsterdam, Amsterdam, The Netherlands

## Background

The 5’ untranslated leader of the HIV-1 RNA genome contains several highly conserved structures that control late virus replication steps. The DIS, SD and Ψ hairpins control the processes of RNA dimerization, splicing and packaging, respectively. Relatively few studies have focused on the short single-stranded domains that connect these hairpin structures. Using the *in vivo* SELEX approach, we randomized these domains in the infectious clone pLAI and selected replication-competent viruses. We thus probed the sequence space that is compatible with HIV-1 replication as an initial test of the function of these three regions. We also analyzed the impact of selected sequences on the secondary structure of the leader RNA, that is the individual hairpin motifs and the proposed LDI:BMH riboswitch that controls multiple leader RNA functions.

## Materials and methods

The domains upstream of the DIS (region 1, nucleotides 237-242), between DIS and SD (region 2, nucleotides 278-281) and between SD and Ψ (region 3, nucleotides 301-305) were randomized in pLAI. These virus DNA libraries were transfected into the SupT1 T cell line and the supernatant was passaged for multiple weeks to select the most replication-competent variants. Interesting variants were recloned into pLAI to confirm the phenotype. The impact on RNA secondary structure was probed *in silico* using RNA secondary structure software.

## Results

Although the sequence of all 3 motifs is highly conserved among natural HIV-1 isolates, quite different results were obtained for these regions. A strict requirement for the wild-type sequence (GCAGGA) was observed for region 1, although two point mutants (GCAGUA and GCAAGA) were also selected. A more relaxed sequence requirement was apparent for region 2, as a larger variety in sequences was selected compared to region 1. In sharp contrast, the highly conserved A5-stretch of region 3 did not reappear in SELEX. None of the selected sequences affected the folding of the individual hairpin motifs or the global LDI:BMH equilibrium of the leader RNA.

## Conclusions

Our results show a strong sequence requirement for the DIS hairpin flanking regions 1 and 2, whereas the sequence of region 3 (between the SD and Ψ hairpins) seems to have a less prominent role. We postulate that the sequences of region 1 and to a lesser extent 2 are important for the binding of specific protein factors that support leader RNA mediated functions. *In silico* analysis indicates that sequences are avoided in all 3 single-stranded domains that affect the local RNA folding of the hairpins or the LDI: BMH riboswitch. Surprisingly, a large variety of region 3 sequences support HIV-1 replication, despite the strong conservation of the A5 stretch in natural isolates. We hypothesize that this may reflect the subtle evolutionary pressure of HIV-1 to acquire an A-rich RNA genome.

